# The Role of Epidermal Growth Factor Receptor in Cancer Metastasis and Microenvironment

**DOI:** 10.1155/2013/546318

**Published:** 2013-08-07

**Authors:** Takamitsu Sasaki, Kuniyasu Hiroki, Yuichi Yamashita

**Affiliations:** ^1^Department of Gastroenterological Surgery, Fukuoka University School of Medicine, Nanakuma 7-45-1, Jonan-ku, Fukuoka 814-0180, Japan; ^2^Department of Molecular Pathology, Nara Medical University, 840 Shijo-cho, Kashihara, Nara 634-8521, Japan

## Abstract

Despite significant improvements in diagnosis, surgical techniques, and advancements in general patient care, the majority of deaths from cancer are caused by the metastases. There is an urgent need for an improved understanding of the cellular and molecular factors that promote cancer metastasis. The process of cancer metastasis depends on multiple interactions between cancer cells and host cells. Studies investigating the TGF**α**-EGFR signaling pathways that promote the growth and spread of cancer cells. Moreover, the signaling activates not only tumor cells, but also tumor-associated endothelial cells. TGF**α**-EGFR signaling in colon cancer cells creates a microenvironment that is conducive for metastasis, providing a rationale for efforts to inhibit EGFR signaling in TGF**α**-positive cancers. In this review, we describe the recent advances in our understanding of the molecular basis of cancer metastasis.

## 1. Introduction

Epidermal growth factor receptor (EGFR) is a key factor in epithelial malignancies, and its activity enhances tumor growth, invasion, and metastasis [1]. EGFR is a member of the ErbB family of tyrosine kinase receptors that transmit a growth-inducing signal to cells that have been stimulated by an EGFR ligand (e.g., TGF*α* and EGF) [2, 3]. In normal tissues, the availability of EGFR ligands is tightly regulated to ensure that the kinetics of cell proliferation precisely match the tissues' requirements for homeostasis. In cancer, however, EGFR is often perpetually stimulated because of the sustained production of EGFR ligands in the tumor microenvironment [4, 5] or as a result of a mutation in EGFR itself that locks the receptor in a state of continual activation [6]. Aberrant expression of TGF*α* or EGFR by tumors typically confers a more aggressive phenotype and is thus often predictive of poor prognosis [7–10]. Not surprisingly, EGFR has emerged as a principal target for therapeutic intervention.

## 2. EGF-Like Ligands and EGFR

Receptor tyrosine kinases (RTKs) are primary mediators of many of these signals and thus determine the fate of the cell: growth, differentiation, migration, or death. The ErbB family of RTKs consists of four receptors: ErbB-1 (EGFR), ErbB-2 (HER2 or Neu), ErbB-3, and ErbB-4 [11, 12]. The mature EGF receptor is composed of a single polypeptide chain of 1186 amino acid residues and a substantial amount of N-liked oligosaccharide. A single hydrophobic membrane anchor sequence separates an extracellular ligand-binding domain from a cytoplasmic domain that encodes an EGF-regulated tyrosine kinase [13–15]. The hallmark of the cytoplasmic protein of this receptor is the sequence defining the tyrosine kinase domain.

Ligand binding induces receptor hemo- or heterodimerization that is essential for activation of the tyrosine kinase. Six mammalian ligands that bind to EGFR have been characterized, including epidermal growth factor (EGF), transforming growth factor-*α* (TGF*α*), amphiregulin, heparin-binding EGF-like growth factor, betacellulin, and epiregulin [16, 17]. Tyrosine kinase activity following ligand binding is essential and is the first step in the EGF signal transduction pathway [18], once the ligand binds the receptor and further stimulates multiple signal pathways including Ras/mitogen-activated protein kinase, phosphatidylinositol 3-kinase/Akt, nuclear factor-*κ*B, and others [19–22].

## 3. Colorectal Cancer and TGF*α*/EGFR Signaling

Studies investigating the signaling pathways that promote the growth and spread of cancer cells suggest that the information transmitted by means of TGF*α*-EGFR signaling is particularly important for progression of tumors that develop in the colon [23–26].

Overexpression of the EGFR and its ligands, TGF*α*, has been correlated with poor prognosis [27–29]. Colon cancer cells secrete TGF*α* in response to hypoxia and the ligand signals, the cell surface EGFR, to initiate a sequence of cell survival programs [30]. This activation of the EGFR signaling pathways stimulates downstream signaling cascades involved in cell proliferation (Ras/mitogen-activated protein kinase [MAPK]) and antiapoptosis (phosphatidylinositol 3-kinase [PI3K]/Akt) [20, 31, 32]. In addition, the overexpression of TGF*α* and EGFR by many carcinomas correlates with the development of cancer metastasis, resistance to chemotherapy and poor prognosis [27, 32, 33].

## 4. Metastatic Colorectal Cancer

The expression levels of TGF*α*, EGF, and EGFR have been shown to correlate with progressive tumor growth, development of metastasis, and resistance to chemotherapy [27, 32, 34]. Measurements of EGFR expressed on human colon cancer cells *in vitro* indicate that metastatic cells may express as much as five-times more EGFR in comparison to nonmetastatic cells [35]. Reports examining the distribution of EGFR and TGF*α* on colorectal biopsies also conclude that the receptor-ligand pair is a characteristic feature of more advanced tumors [27, 36–38].

## 5. Microenvironment of Colon Cancer for Metastasis

The concern of the microenvironment of tumors has been growing. The process of cancer metastasis is sequential and selective and contains stochastic elements. The growth of metastases represents the endpoint of many lethal events that few tumor cells can survive. Angiogenesis refers to the development of new blood vessels from the preexisting vasculature. Angiogenesis plays a key role in the initiation of metastases. Tumor cell proliferation and survival depend on the vasculature to supply adequate oxygen and nutrients [39]. The extent of angiogenesis depends on the balance between proangiogenic and antiangiogenic factors released by tumor cells and host cells [40, 41]. The communication networks that are established between tumor cells and the nonneoplastic cells in the microenvironment of primary tumors play a critical role in tumor growth and development of metastasis [42, 43].

Data derived from examinations of human lung cancer brain metastases indicate that tumor cell division takes place within 75 *μ*m of the nearest blood vessel, whereas tumor cells residing beyond 150 *μ*m from a vessel undergo programmed cell death [44]. The turnover rate of endothelial cells within the tumor-associated vessels is 20 to 2,000 times faster than the rates of the vessels in normal organs [45]. One recent detailed study of the multiple clinical specimen of human neoplasms reported that proliferation rate of endothelial cells within the vasculature of normal human organs has been reported to be <0.01%, whereas 2% to 9% of endothelial cells in tumor-associated vessels divide daily [46].

**Figure 1:**
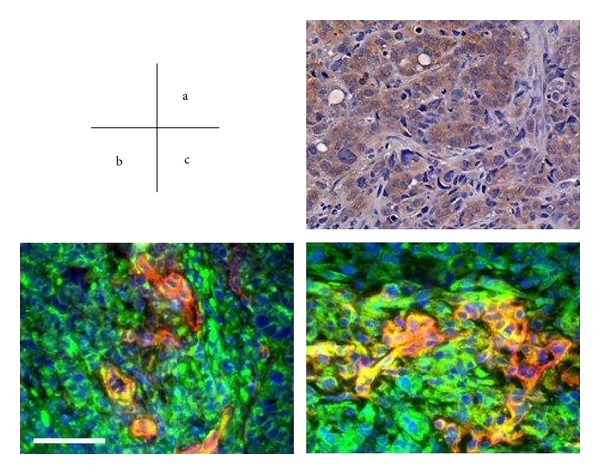
Immunohistochemical analyses of expression of TGF*α*, EGFR, and phosphorylated EGFR on tumor cells and tumor-associated endothelial cells in orthotopically implanted colon tumors. (a) TGF*α* expression in the tumor cells. (b) EGFR was present on tumor cells (green) and was also detected on the tumor-associated vasculature (yellow). (c) Expression of phosphorylated EGFR was localized to both tumor cells (green) and the supporting vascular network (yellow). Scale bars = 100 *μ*m [50].

Expression of EGF, VEGF, or their respective receptors has been shown to correlate with angiogenesis and progressive growth of human carcinomas of the colon [47]. Furthermore, the expression of EGFR, VEGFR, and the phosphorylated receptors was observed on tumor-associated endothelial cells. These receptor and phosphorylated receptor were expressed on tumor-associated endothelial cells only when the tumor cells expressed the relevant ligands. These findings suggest that ligands released by tumor cells can upregulate the expression of receptors on tumor-associated endothelial cells in a paracrine manner [48–50] (Figure 1).

**Figure 2:**
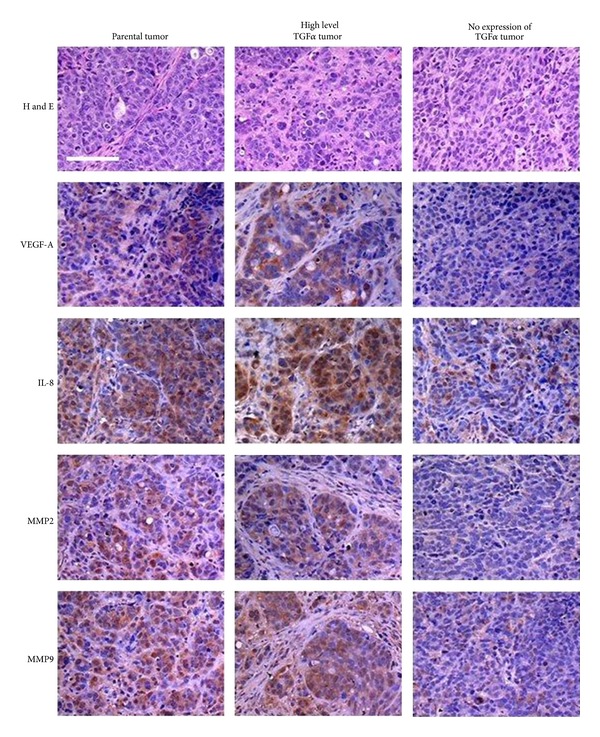
Immunohistochemical analyses of expression of VEGFA, IL-8, MMP-2, and MMP-9 in orthotopically implanted colon tumors. The parental colon cancer cell line originates from a primary human colon carcinoma. The clones were expanded, and the resulting populations were screened for production of TGF*α*. The microenvironment of selected high level TGF*α* tumors is enriched in VEGFA, IL-8, MMP-2, and MMP-9. Expression of the angiogenic proteins in tumors that do not express TGF*α* is significantly attenuated. Scale bars = 100 *μ*m [50].

The angiogenic proteins, VEGFA and IL-8, were strongly expressed in the microenvironment of tumors that produced TGF*α*. In contrast, expression levels of VEGFA and IL-8 were considered unremarkable in TGF*α*-deficient tumors. VEGFA is often regarded as the prototypical angiogenic protein in that it can stimulate each of the cellular responses required for the generation of a new vascular bed (e.g., migration, proliferation, protease production, and cell survival) [51, 52]. There are also several lines of evidence suggesting that some cells rely on TGF*α*-induced stimulation of EGFR to enhance their production of IL-8. These data demonstrate that the extensive EGFR network (autocrine and paracrine) generated by TGF*α*-expressing colon cancer cells leads to a greater production of proangiogenic proteins (TGF*α*, VEGFA, and IL-8) in the microenvironment of primary tumors (Figure 2).

Several other factors that promote angiogenesis and tumor cell invasion were also preferentially expressed in the microenvironment of TGF*α*-positive tumors. Specifically, we noted robust expression of two members of the MMP family, MMP-2 and MMP-9, in tumors that were positive for TGF*α* [50] (Figure 2). These proteolytic enzymes perform several key functions during angiogenesis (e.g., increase the bioavailability of angiogenic proteins, degrade basement membrane barriers, and promote endothelial cell migration) and metastasis (e.g., invasion and extravasation) [53].

**Figure 3:**
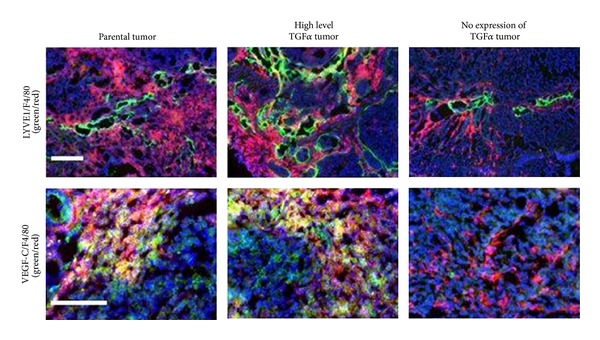
Immunofluorescent staining of LYVE-1, F4/80, and VEGFC in human colon carcinoma cells expressing different levels of TGF*α*. Lymphatic vessels are labeled with LYVE-1 (green) and macrophage cells with F4/80 (red). The number of tumor-associated lymphatic vessels was greatest in selected high-level TGF*α* tumors and fewest in tumors that do not express TGF*α*. Tumor recruitment of macrophages was also fewest in tumors that do not express TGF*α*. Macrophage cells localized to selected high level TGF*α* tumors also expressed LYVE-1. The macrophage population recruited to TGF*α*-expressing tumors also produced abundant levels of the lymphatic endothelial cell growth factor VEGFC. Scale bars = 100 *μ*m [50].

Macrophages are also capable of creating structural and biochemical imbalances in the extracellular matrix. A closer inspection of the tumor-infiltrating macrophages in TGF*α*-positive tumors showed that these cells express the lymphangiogenic growth factor VEGFC. The few macrophages present in the TGF*α*-negative tumors in our study did not express VEGFC, but they did so when tumor cells were transfected with TGF*α* transgenes and then implanted into the cecal walls of mice [50] (Figure 3). These results add to the growing evidence that suggests that macrophages are a major source of VEGFC in pathological tissues and, therefore, function as central regulators of the lymphatic vascular surface area [54, 55].

**Figure 4:**
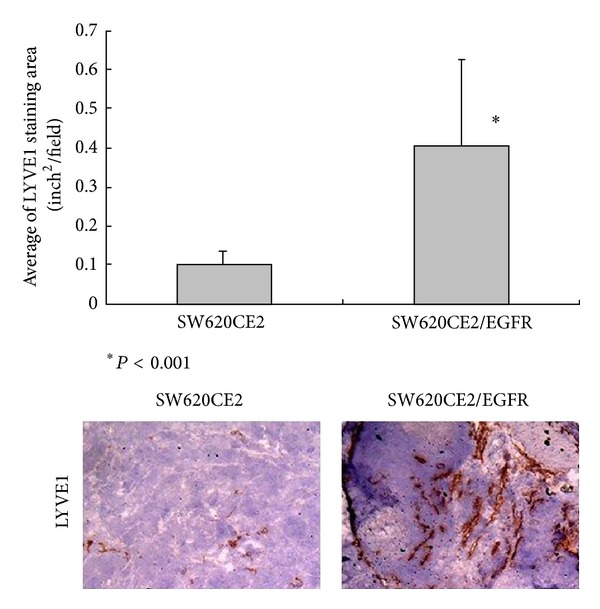
Mean density of LYVE-1 on orthotopic colon tumors expressing different levels of EGFR. The SW620CE2 is human colon cancer cell line. SW620 cells were injected into the cecal wall of nude mice. Three months after the injection, cecal tumors were harvested. Cells were established in culture. Primary cultures were passaged *in vitro* two or three times, and then, cells were injected into the cecum of another set of nude mice. The selection cycle was repeated two times to yield cell lines designated SW620CE2. SW620CE2 did not produce detectable levels of EGFR. SW620CE2/EGFR was established from SW620CE2 which was transfected sense EGFR plasmids. Cells (5 × 10^5^) in 50 *μ*L of Hanks' buffered saline solution were injected into the cecal wall of nude mice. The number of lymphatic vessels in SW620CE2/EGFR tumors was fourfold higher than that observed in SW620CE2 tumors [56].

The number of tumor-associated lymphatic vessels in the different tumors was determined by counting the number of vessels that were positive for LYVE-1. LYVE-1 is an integral membrane protein that functions as the receptor for the glycosaminoglycan hyaluronan. LYVE-1 is also expressed by sinusoidal endothelial cells in the liver and spleen and by some macrophages [32]. We found that the number of lymphatic vessels in EGFR-expressing tumors was fourfold higher than that observed in EGFR-deficient tumors, demonstrating that TGF*α*-EGFR signaling is an important cofactor for expansion of the tumor-associated lymphatic vascular network [56] (Figure 4).

Supportive evidence for the involvement of TGF*α* in metastasis comes from a recent study that identified TGF*α* as a member of the gene set that that identifies colorectal cancer cells that metastasize to the liver [57]. Alternatively, it has been known for some time now that a high vascular density increases the likelihood that tumor cells will enter the systemic circulation and reach distal organs of metastasis [58], and we found that the activation of autocrine and paracrine TGF*α*/EGFR signaling networks affects the tumor microenvironment in colon cancer and determines its impact on the formation of metastases.

## 6. Microenvironment of Biliary Tract Cancers for Metastasis

Biliary tract cancers express EGFR in 60.7% of cases [59]. The EGFR-overexpressing gallbladder cancer (GBC) cases show poorly differentiated histology and decreased survival of 1.5 years in median survival [60]. Amplification and point mutations of the EGFR gene have been reported to be 1% and 15%–26.5%, respectively, in GBC [61–63]. The HGF receptor c-Met is involved in the early carcinogenesis of biliary tract cancers [64]. c-Met is expressed in 74% of invasive GBC and is associated with invasive depth [65]. Because HGF is secreted from fibroblasts, c-Met activation depends on the cancer-host interaction [66]. Transforming growth factor-b is widely expressed in advanced GBC and is associated with angiogenesis and tumor-associated macrophage infiltration as well as with stromal fibrosis [67, 68]. Epidermal growth factor receptor, c-Met, and TGF-b have recently been implicated in the process of epithelial-mesenchymal transition (EMT) [69–71]. EMT comprises a switch in cell differentiation from polarized epithelial cells to contractile and motile mesenchymal cells [72]. In EMT-type cells, the reduction of the epithelial marker E-cadherin (ECD) occurs in parallel with the induction of the mesenchymal marker vimentin (VIM) [73]. EMT occurs during cancer progression and enhances invasion and metastasis [72].

## 7. Strategy of Treatment

Inhibiting signaling pathways through EGFR represents a good strategy for therapeutic intervention. Gefitinib inhibits EGF-stimulated EGFR autophosphorylation in a broad range of EGFR-expressing human cancer cell lines [74]. Cetuximab, a monoclonal antibody targeting EGFR, has been shown to induce apoptosis of colorectal cancer cells [75–77]. TGF*α*-EGFR signaling in both tumor-associated endothelial cells and the tumor cells themselves is important in the progression of colon cancer. Abrogating the signaling activation by a dual tyrosine kinase inhibitor in combination with conventional therapy can induce a significant decrease in proliferation of tumor cells and significant apoptosis of both tumor cells and endothelial cells. Targeting the EGFR and VEGFR signaling in tumor vasculature with antineovascular therapy provides a new approach to the treatment of colon cancer.

In cholangiocellular carcinoma cell lines, the anti-EGFR antibody cetuximab is partially effective in EGFR-expressing cells [78]. KRAS mutations affect the efficacy of cetuximab in these cells. Gefitinib, a selective EGFR tyrosine kinase inhibitor, inhibits the phosphorylation of EGFR, ERK, and AKT and induces G1 arrest and apoptosis by upregulating p21 and p27 and BAX activation in GBC cells [79]. Epidermal growth factor receptor targeting is, therefore, critical in the treatment of GBC.

## 8. Conclusion

The activation of TGF*α*-EGFR signaling in primary colon tumors contributes to the spread of tumor cells to lymph nodes and the liver. TGF*α*-expressing tumors cells are more proficient in their ability to initiate metastases by virtue of their ability to communicate with the resident nontumor cell population. Therapeutic interventions that are designed to block EGFR signaling in TGF*α*-positive colon tumors will likely have a negative impact on a number of processes that are essential for metastasis formation.

## Abbreviations

EGF: Epidermal growth factor
EGFR: Epidermal growth factor receptor
TGF*α*: Transforming growth factor-*α*
RTKs: Receptor tyrosine kinases
VEGF: Vascular endothelial growth factor
VEGFR: Vascular endothelial growth factor receptor
IL-8: Interleukin-8
MMP: Matrix metalloproteinase
LYVE-1: Lymphatic vessel endothelial hyaluronate receptor 1
GBC: Gallbladder cancer
EMT: Epithelial-mesenchymal transition
ECD: E-cadherin
VIM: Vimentin.
